# Bone Density Changes Following Radiotherapy to Vertebral Metastases

**DOI:** 10.7759/cureus.15417

**Published:** 2021-06-03

**Authors:** Garrett L Jensen, Ravi Gaddipati, Kendall P Hammonds, Andrew Morrow, Gregory P Swanson

**Affiliations:** 1 Radiation Oncology, Baylor Scott & White Health, Temple, USA; 2 Radiation Oncology, Texas A&M University College of Medicine, Temple, USA; 3 Biostatistics, Baylor Scott & White Health, Temple, USA

**Keywords:** bone density, vertebrae, spine, insufficiency fracture, bone healing

## Abstract

Introduction

Patients have increasing longevity and time for bone healing following radiotherapy (RT) for treatment of bone metastases (BM). Attempts to assess the treatment response of bone metastases have been either limited or heavily subjective. Our goal was to try to quantitate cancer-involved bone changes after RT using changes in bone mineral density (BMD) from computer tomographic (CT) imaging.

Methods

Retrospectively, 117 spinal metastases were identified that received RT with follow-up CT scans >9 months following CT simulation. Contoured volumes included: the metastasis (gross tumor volume; GTV); the involved vertebra (gross bone volume; GBV); a total lytic volume (Lyt); a dominant lytic volume (Domlyt); a control volume, and the nearest uninvolved, unirradiated vertebra (control bone volume; CBV). The Hounsfield-density calibration curve was used to measure the density of these volumes before and after treatment.

Results

Whether using raw or control-adjusted changes, the absolute and percent change in density of the GBV, GTV, Lyt, and Domlyt volumes all significantly increased (each p<0.0001). The increase in the density of Domlyt volumes was greater than that of Lyt volumes (p=0.0465), which were greater than GTV (p=0.0065), which were greater than GBV (p<0.0001). On multivariate analysis, only the biologically effective dose (BED) dose significantly correlated with GTV density change (p=0.0175). K means clustering created groups by initial lesion size, GTV, or GBV density. A significant difference in GTV density change was not detected between any groups.

Conclusion

Increases in BMD are associated with healing regardless of lesion size or initial density. A prospective study to determine whether long-term control is related to early density measurements is needed.

## Introduction

Metastatic cancer to the bone has been treated with radiation for over 100 years. Most treated patients have a metastatic disease with correspondingly short survival. In addition, with the primary goal usually being subjective (pain relief), there has been a minimal indication for follow-up imaging. As a result, in spite of many thousands of patients having been treated, there is surprisingly little data on bone changes after treatment. Radiation is thought to delay bone healing due to locally maladaptive effects on perfusion and the suppression of osteoclasts and osteoblasts (plus associated mesenchymal cells) as it does with any proliferating cells. In contradistinction, more certain is that bone containing cancer cannot heal. As most radiation oncologists have observed, radiation can obliterate cancer and the bone will heal. For patients with lytic lesions, healing can be easily determined observationally (subjectively). More problematic is the majority of patients who have blastic or mixed blastic/lytic lesions that are much more difficult to measure. As a result, the current guidelines for assessing treatment response in metastatic disease usually exclude those patients. For example, Response Evaluation Criteria in Solid Tumors (RECIST) criteria are limited to lytic lesions with a measurable tumor mass of at least 1 cm [1]. There is a significant need for a reproducible way to measure bone response.

The universal standard recognized mode for the evaluation of bone integrity is bone density. It is not difficult to conceptualize that with a lytic lesion, as the bone heals, the density will return closer to normal. With blastic and mixed lesions, how that should look is less obvious. With purely blastic lesions, which are already denser than normal bone, the possibilities are the density increases as the bone heals, decreases as the bone remodels to a more normal state, or it all balances out with no appreciable change. The larger the lytic component, the more likely the overall density will increase. Computed tomographic (CT) imaging can assess cortical and trabecular bone with high resolution, show reossification, and Hounsfield units (HU) are highly correlated with bone mineral density (BMD) [2-3]. Our goal was to try to quantitate and define how cancer involved bone changes after treatment for each of the disparate states of presentation (blastic, lytic, or mixed) in the spine based on CT.

## Materials and methods

The spine was chosen for analysis given the relative structural uniformity anatomically with the goal of making the findings more consistent and reproducible. With institutional review board approval, we performed a retrospective review of all the patients treated for bone metastases from May 2008 to November 2017. Over 600 patients were identified. Of those, approximately 200 were treated for spinal metastases. Imaging data were then collected. It became evident early on that there was a paucity of imaging in the post-treatment six to nine months, primarily due to patient death. Since the treated bone was not specifically re-imaged, the availability of imaging depended on whether the patient lived long enough to be restaged. Therefore, imaging only became routinely available after nine months. We, therefore, collected all post-treatment imaging, analyzing the one closest to the one-year anniversary of treatment with a median follow-up time of 14 months (range, 9-25 months). A comparison was made with the radiation planning CT, which was available in all patients. With these criteria, 117 vertebrae in 99 patients had follow-up imaging after radiation treatment for spinal metastases.

**Figure 1:**
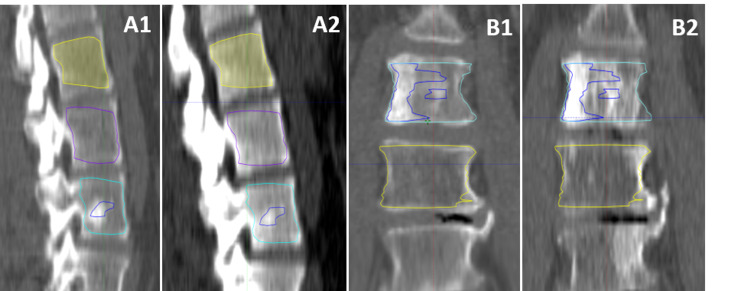
Delineating lytic portions of vertebral metastases Metastatic breast cancer to L4 treated with 3000 cGy in 10 at A) CT simulation scan and B) 16-month follow-up scan. Included volumes: GTV, gross tumor volume; GBV, gross bone volume; CBV, control bone volume; Domlyt, dominant lytic volume; Lyt, total lytic volume (consisting of all obviously lytic components of the GTV, including the Domlyt volume).

**Figure 2:**
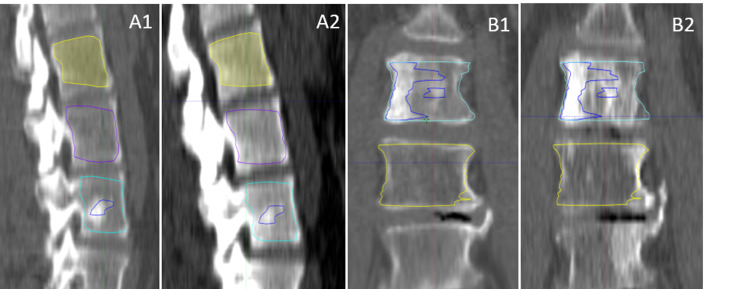
Delineating volumes of diseased and control vertebrae Metastatic prostate cancer to L3 treated with 20 Gy in one fraction at CT simulation scan (A1) and at the 16-month follow-up (A2); Metastatic breast cancer to L4 treated with 30 Gy in 10 fractions at CT simulation scan (B1) and at the 24-month follow-up (B2). The control bone volume (CBV), normal irradiated bone volume (NBV), gross irradiated bone volume (GBV), and gross tumor volume (GTV) are delineated by the yellow, purple, light blue, and dark blue contours, respectively.

The entirety of the involved vertebrae was contoured into the inner third of the denser cortex and endplates, designated gross bone volume (GBV), with a planning system resolution of 5 mm. The visually apparent lesion(s) were contoured as gross tumor volume (GTV). The GTV was the sum of all contoured volumes. Sub-volumes to further characterize the GTV lesions included, when present, a total lytic volume (Lyt), consisted of all obviously lytic (radiolucent) components of the GTV, as well as a dominant lytic volume (Domlyt), which was the largest isolated lytic component of the GTV (Figure 1). A control bone volume (CBV) was measured for the nearest disease-free vertebrae of the same type (cervical, thoracic, lumbar) that were entirely outside of the 50% isodose line of the radiation treatment field to account for normal density changes over time (Figure 2). If such a control bone could not be used or developed disease on follow-up scan (n=24, 20.5%), a virtual control density was used by interpolating density data based on age (at the time of each scan) and vertebral level from Patel et al. [4]. As an example, to calculate a control-adjusted absolute change in density for the GBV, we would use the formula: (post-RT GBV density / post-RT CBV density) - (pre-RT GBV density / pre-RT CBV density). To calculate a control-adjusted percent change for the GBV, we would use the formula: ([post-RT GBV density/post-RT CBV density] - [pre-RT GBV density/pre-RT CBV density]) / (pre-RT GBV density/pre-RT CBV density)).

All volumes were drawn using Varian Contouring (Version 13.6, Varian Medical Systems Inc., Palo Alto, California). To analyze the effect of radiotherapy (RT) on BMD of the aforementioned volumes over time, CT simulations were registered to follow-up scans using Varian Image Registration. Volumes were copied from the CT simulation scan to the follow-up scan using rigid transformation to the vertebrae of interest as well as one to three proximal and/or distal vertebrae. Subsequent manual shifts of volumes were allowed at the physician’s discretion.

Radiographically, some tumors appeared obviously/predominantly lytic or blastic, and many were mixed. However, instead of creating groups based on subjective visual inspection, we created groups using objective density and volume. The simplest approach was to analyze the entire vertebrae (GBV), but since these results are easily skewed by the tumor volume (i.e. a very small tumor has minimal effect on the overall vertebrae density), we also analyzed the results as per the lesions themselves (GTVs). Patients were partitioned by initial GBV or GTV density, using K-means clustering. Finally, we excluded the high-density GTV and GBV groups due to the paucity of numbers to create initial clusters by lesion size proportional to the vertebrae (GTV volume/GBV volume). Bone density was considered to be stable or increased if the change in density was >-0.05 g/cm^3^.

Since the relationship between HU and density is non-linear, a script was developed to derive density (g/cm^3^) from HU. A CT scanner (GE Optima 580; GE Healthcare, Chicago, Illinois) calibrated to American College of Radiology (ACR) standards was used to image masses of known density (CIRS Electron Density Reference Phantom, Model 62; CIRS, Norfolk, VA). The reported HU for each known density was used to construct a calibration curve. The reported density reflects the average of converted HU within the outlined region of interest.

The biologically effective dose (BED) was calculated using an alpha/beta ratio of 3. Statistical analysis was done using SAS version 9.4 (SAS Institute Inc., Cary, NC). Wilcoxon signed-rank tests were used to assess significant change from pre- to post-radiation. Wilcoxon rank-sum tests were used to assess the difference in change between the two groups. A Kruskal-Wallis test was used to assess the difference in change between three or more groups. Linear regressions were used to assess the multivariate relationships between variables and continuous variables. Statistical significance was set at p≤0.05.

## Results

Only one patient had radiologic progression of disease in the treated vertebrae more than nine months after treatment and was thus precluded from the analysis. Overall, 117 vertebrae were analyzed. Of those, GBV density was stable in 35 (29.9%) vertebrae and increased in 72 (61.5%) vertebrae. GTV density was stable in 26 (22.2%) vertebrae and increased in 79 (67.5%) vertebrae. A Domlyt volume was drawn in 93 (79.5%). Domlyt density was stable in 18 (19.4%) vertebrae and increased in 75 (80.7%) vertebrae. A Lyt volume was drawn in 96 (82.1%). Lyt density was stable in 19 (19.8%) vertebrae and increased in 73 (76.0%) vertebrae.

See Table 1 for patient characteristics. The change in density following radiation was only significantly affected by GTV volume (p=0.0009, correlation coefficient 0.3026), BED dose (p=0.0018, correlation coefficient -0.2861), and use of androgen ablation during the follow-up period (p=0.0232). On multivariate analysis, only the BED dose remained significant (p=0.0175), with each unit of Gy 3 increase resulting in a 0.0007 decrease in the magnitude of GTV density increase.

**Table 1:** Patient characteristics GTV, gross tumor volume; BED, biologically effective dose at 3 Gy ^a^Interval between CT simulation scan for radiation treatment planning and CT scan following radiation treatment ^b^Used immediately prior to, during, or in the follow-up period after radiotherapy

Continuous Variables			Change in GTV density (g/cm^3^)	
	Median (range)		Spearman Correlation	p-value
Age (years)	64.9 (33.3-99.0)		-0.0406	0.6637
GTV (cc)	7.2 (0.24-38.8)		0.3026	0.0009
Follow-up Time (months)^a^	14.0 (9-25)		0.0398	0.6698
Dose (Gy)	30 (8-45)		0.0591	0.5268
Fractions	10 (1-15)		0.1109	0.2340
BED (Gy_3_)	60.0 (29.3-216.0)		-0.2861	0.0018
Discontinuous Variables		N (%)	Median (range)	
Primary:	Breast	42 (35.9)	0.07 (-0.25-0.38)	0.0530
	Other	26 (22.2)	0.02 (-0.13-0.35)	
	Prostate	24 (20.5)	0.09 (-0.15-0.5)	
	Lung	15 (12.8)	-0.01 (-0.12-0.2)	
	Myeloma	10 (8.6)	0.04 (-0.02-0.49)	
Gender	Male	51 (43.6)	0.06 (-0.15-0.5)	0.1363
	Female	66 (56.4)	0.05 (-0.25-0.49)	
Race	Caucasian	89 (76.1)	0.05 (-0.18-0.49)	0.7172
	African American	20 (17.1)	0.04 (-0.12-0.23)	
	Other	8 (6.8)	0.13 (-0.25-0.50)	
Androgen Ablation^b^	Yes	20 (17.1)	0.1 (-0.15-0.5)	0.0232
	No	97 (83.0)	0.04 (-0.25-0.49)	
Denosumab/Bisphosphonates^b^	Yes	56 (47.9)	0.05 (-0.18-0.5)	0.6272
	No	61 (52.1)	0.05 (-0.25-0.35)	
Vertebral Level	Cervical	9 (7.7)	.15 (-0.25-0.28)	0.0971
	Thoracic	60 (51.2)	0.03 (-0.18-0.35)	
	Lumbar	48 (41.0)	0.05 (-0.15-0.5)	

**Figure 3:**
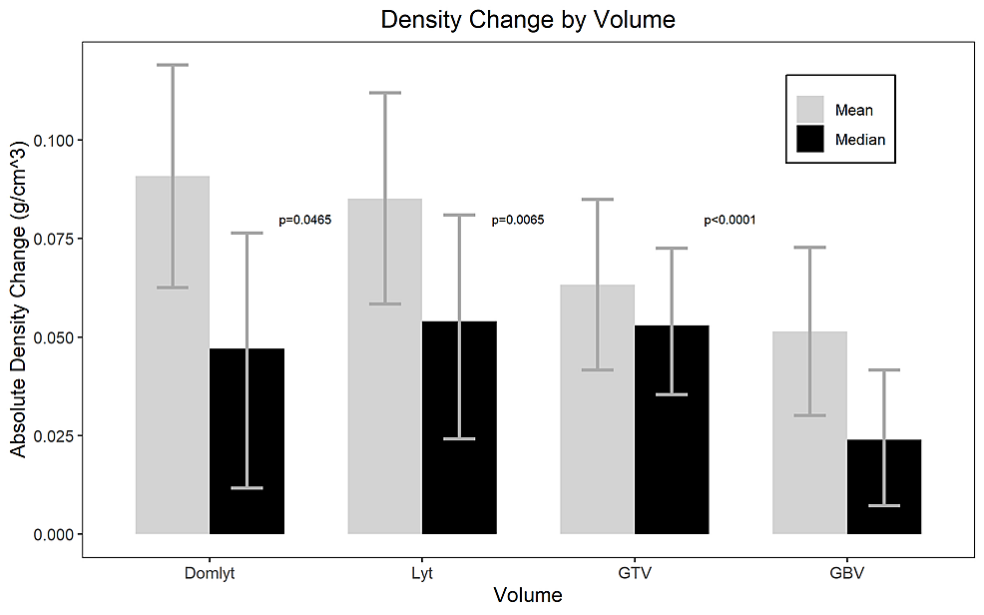
Density change by volume Absolute density change in g/cm^3 ^of the dominant lytic volume (Domlyt); total lytic volume (Lyt), gross tumor volume (GTV), and gross bone volume (GBV) Wilcoxon signed-rank p-values represent the significance of density change between adjacent volumes. Error bars represent the 95% confidence interval for mean values and bootstrapped 95% confidence intervals for median values.

Whether using raw or control-adjusted changes, the absolute and percent change in density of the GBV, GTV, Lyt, and Domlyt volumes all significantly increased throughout our population (each p<0.0001). See Table 2. By absolute change in density, Domlyt volumes increased density in density more than Lyt volumes (p=0.0465), Lyt volumes increased in density more than GTV (p=0.0065), and GTV increased in density more than GBV (p<0.0001). See Figure 3. These changes remained significant with analysis of percent change.

**Table 2:** Density change by volume following radiotherapy GBV, gross bone volume; GTV, gross tumor volume; Lyt, lytic component of GTV; Domlyt, largest solitary lytic component of GTV

	GBV	GTV	Lyt	Domlyt
Pre-RT density, g/cm^3^, median (range)	1.18 (1.1-1.6)	1.19 (1.0-2.2)	1.10 (1.0-1.5)	1.09 (1.0 - 1.5)
Absolute density change, g/cm^3^, median (range)	0.02 (-0.14-0.58)	0.05 (-0.25-0.5)	0.05 (-0.12-0.83)	0.05 (-0.05-0.89)
p-value	<0.0001	<0.0001	<0.0001	<0.0001
Control adjusted absolute density change, g/cm^3^, median (range)	0.02 (-0.18-0.49)	0.04 (-0.24-0.47)	0.04 (-0.15-0.77)	0.04 (-0.14-0.83)
p-value	<0.0001	<0.0001	<0.0001	<0.0001
Density change (%), median (range)	2.1 (-10.6-51.7)	4.2 (-17.4-45.5)	4.4 (-9.1-77.0)	4.0 (-4.8-83.7)
p-value	<0.0001	<0.0001	<0.0001	<0.0001
Control adjusted density change (%), median (range)	1.7 (-18.5-48.9)	3.4 (-20.3-47.4)	4.4 (-15.3-82.4)	3.8 (-15.0-89.3)
p-value	<0.0001	<0.0001	<0.0001	<0.0001

K-means clustering using GTV or GBV each resulted in the creation of three groups. The highest density group using clustering by GTV and GBV contained only three or nine patients, respectively. A significant difference in density change was detected only when clusters were created by GTV/GBV volume with regard to raw GBV density change (p=0.0075). See Table 3. The median GTV percent change of the low, middle, and high-density GTV groups was 4.3, 4.2, and -1.5%, respectively. The median GBV percent change of the low, middle, and high-density GBV groups was 2.3, 1.5, and 1.3%, respectively. The median GBV percent change of the small, medium, and large lesion groups was 0.9, 0.4, and 5.2%, respectively.

**Table 3:** Density changes clustered by initial characteristics RT, radiotherapy; GBV, gross bone volume; GTV, gross tumor volume; Lyt, lytic component of GTV; Domlyt, largest solitary lytic component of GTV *Significance did not vary using absolute density change or control adjusted percent change. **Only three patients were in this subgroup. Thus, the comparative statistical analysis is limited to the lowest and medium-density groups.

			GBV		GTV		Lyt		Domlyt	
Clusters	Subgroups	Pre-RT density, g/cm3, median (range)	Density Change (%)	P-value*	Density change (%)	P-value*	Density Change (%)	P-value*	Density Change (%)	P-value*
GTV	Lowest density	1.14 (1.05-1.26)	1.2 (-5.8-51.7)	0.7251	4.3 (-8.7-45.5)	0.3228	4.0 (-4.9-77.0)	0.2017	3.8 (-4.8-83.7)	0.7219
	Medium density	1.34 (1.28-1.57)	3.4 (-10.6-28.5)		4.21 (-17.43-28.5)		5.13 (-9.10-18.89)		4.45 (-2.83-30.31)	
	Highest density	2.04 (1.87-2.15)	0.61 (-2.76-1.31)	N/A**	-1.45 (-3.47-3.82)	N/A	N/A	N/A	N/A	N/A
GBV	Lowest density	1.14 (1.0-1.6)	2.3 (-5.8-51.7)	0.8381	4.37 (-13.4-45.5)	0.2737	4.44 (-9.1-77.0)	0.2601	5.11 (-4.8-83.7)	0.5093
	Medium density	1.27 (1.2-1.4)	1.5 (-10.6-46.5)		3.71 (-17.4-28.6)		3.67 (-6.7-18.9)		3.17 (-2.8-30.3)	
	Highest density	1.46 (1.3-1.6)	1.3 (-7.4-13.4)		3.82 (-8.2-13.4)		7.31 (-2.7-11.4)		4.0 (-2.8-10.8)	
GTVcc/ GBVcc	Smallest size	1.19 (1.1-1.3)	0.88 (-10.6-46.5)	0.0075	1.34 (-17.4-28.6)	0.1544	2.13 (-6.7-22.2)	0.1224	2.26 (-4.2-21.9)	0.3333
	Medium size	1.15 (1.1-1.3)	0.35 (-5.6-51.7)		4.5 (-13.4-45.5)		3.70 (-9.1-51.6)		6.38 (-2.8-51.6)	
	Largest size	1.17 (1.0-1.6)	5.18 (-8.3-43.9)		4.5 (-9.2-43.0)		7.19 (-4.9-77.0)		5.84 (-4.8-83.7)	

## Discussion

Until about 10 years ago, follow-up imaging of local bone treatment was not routinely obtained. The exception was in the research realm, where there is great interest in being able to determine bone response. There has been great dissatisfaction in the use of visual/subjective criteria, but there have not been readily available substitutes. As noted previously, the widely adopted RECIST criteria can only be used to evaluate bone if there is a lytic lesion with a soft tissue component greater than 1 cm. Interest in determining the treatment outcome of bone has been more recently spurred by the surge in focal high-dose radiation (stereotactic body radiation/SBRT) and the desire to determine the most effective dose and fractionation schedule. Universally, the standard determination of bone structural integrity is by measuring bone density. The mechanics of using bone density to define bone healing (a return to normal) have yet to be optimized.

Currently, the measurement of bone response is evolving. For example, for the spine, a consensus committee (calling themselves the spine response assessment in neuro-oncology (SPINO) working group) [5] drafted recommendations for response evaluation of the bone. They cited difficulty in determining bone structure with CT and did not discuss the use of density. Their conclusion was that the only widely applicable approach was MRI. Unfortunately, given the significantly higher expense of MRI over CT scan, this is unlikely to be widely applicable and then only in the funded research setting.

Others have tried to evaluate treatment outcomes based on bone density using CT scans, some after radiation [6-17], and others after chemotherapy [18] or bisphosphonates [19]. The radiation studies have been limited by few patients, short follow-up, and high patient drop-off due to death. Most contoured a region of interest (ROI), which included only a portion of gross disease, and did not use similar areas of bone in the patient as controls.

Some of these studies reported outcomes for lesions subjectively grouped as osteolytic, osteoblastic, or mixed [6-9,13,17]. These studies uniformly showed an increase in density after radiation. Also, with the exception of one study in which osteolytic and osteoblastic lesions were markedly underrepresented [8], these studies indicated that increased density following radiation trended toward or was significantly greater in magnitude as the lytic component of lesions increased across different histologies and lesion sites. Bone pain, opioid consumption, and quality of life have all been demonstrated to be inversely correlated with bone density. Correspondingly, patients with osteolytic lesions seem to derive the most benefit from radiation [7,16]. Still, when looking at whether pain response linked with a greater change in density or type of neoplasm, the evidence is mixed [12,14]. We did not show a statistically different change in density based on how ‘lytic’ the GTV or GBV were prior to RT using cluster analysis. However, the most lytic areas of each lesion, represented by the Lyt and Domlyt volumes, had a significantly greater increase in density than the GTV or GBV volumes. The proportional size of the lesions also did not affect density change.

There is evidence that this increase in density following radiation can be accelerated with bisphosphonates [9,19]. Wang et al. reported that chemotherapy prior to RT decreased the magnitude of increased density at all time points following RT while others have reported increased mineralization with the addition of chemotherapy [13,18]. Altered radiation dosing has also shown some evidence for altering bone healing [11,20]. Koswig and Budach found a trend towards increased rates of recalcification in all histologies when comparing patients who received 30 Gy in 10 fractions to patients who received 8 Gy in one fraction. Immediately after radiation, the density appeared to take a bigger ‘hit’ in the more protracted and higher-dose course but rapidly and durably increased thereafter. After an initial decrease from a baseline 17% greater in magnitude, the percent change from baseline at six weeks, three months, and six months was greater in the 30 Gy group than the 8 Gy group by 22%, 33%, and 53%, respectively. If confirmed in a larger study, this difference between groups could be due to a differential effect on tumor cell kill or on surrounding osseous and vascular structures. We did not demonstrate a difference in bone healing for patients on bisphosphonates and/or denosumab, but we did find a weakly negative effect of BED dose on follow-up BMD.

The potentially increased rates of local control with higher ablative doses may be limited by increased rates of fracture [20-21]. Interestingly, we found that an increased BED resulted in a decreased magnitude of density recovery of the GTV following radiation. A variety of factors can contribute an increased risk of fracture following radiation to a metastatic lesion. Chief among them is the loss of structural integrity caused by the invasion and subsequent recession or scarring of the tumor mass. However, another concern is the detrimental effect that radiation can have on portions of bone without the disease. Although others have not reported a change [10,22], Wei et al. found that patients treated with abdominal radiation had a BMD loss that persisted in patients after 12 months with a significant relation to radiation point dose in the vertebrae [23]. In patients treated for bony metastases, Wachenfield et al. also found decreased BMD in normal bone outside of the RT field. Reinbold et al. actually found that the normal bone surrounding the osteolytic site increased in density zero and three months after RT [12]. There has been some suggestion that even bone outside of the radiation field may have its density impacted due to effects on bone marrow stem cells [24]. However, this has not been a consistent finding [8,10,17,23].

Density in these studies was reported in Hounsfield units. It should be noted that a 20% increase in Hounsfield units would only be a 2%-5% increase in BMD (g/cm^3^). See Table 4. In general, the lytic lesions had a greater increase in density, but even the blastic lesions increased, as in our experience. Unfortunately, these authors had the same problem we had, which is that patients that had progression likely died before re-imaging. Overall, the density of diseased bone significantly increased after radiation in patients without subjective radiographic failure. However, some patients still had a decrease of BMD of the GTV or GBV. Lytic portions of disease more reliably increased in density, but there were still exceptions.

**Table 4:** Literature review of density changes after radiotherapy using computed tomographic imaging RT, radiotherapy; ROI, region of interest; GTV, gross tumor volume; IMRT, intensity-modulated radiotherapy; 3dCRT, three-dimension conformal radiotherapy; SBRT, stereotactic body radiotherapy; SF, single fraction; MF, multiple fractions ^a^Groups created by initial visual inspection ^b^Patients given monthly ibandronate

Author	Year	RT treatment (Total Gy/# of Fractions)	Patients at first f/u (n)	Bone type	Primary	Area/volume of reported density	Subset analysis	Lesion Type	Months after RT (% change)			
									0-1	1-3	4-6	7-10
Koswig & Budach [11]	1999	30/10 or 8/1	55 and 52	mixed	mixed	ROI	8Gyx1	mixed	-8	2	10	20
							3Gyx10		-25	24	43	73
Reinbold et al. [12]	1989	40/20	19	spine	mixed	ROI	complete pain relief	lytic	-25	61	N/A	
							no relief		-5	14.1		
Wachenfield et al. [13]	1996	30-36/10-12	14	spine	breast	ROI	by lesion^a^ type	lytic	14	32	51	N/A
								mixed	-1	0	3	
								blastic	0	3	-11	
								normal bone	-33	-35	-42	
Vassiliou et al. [15]	2010	30-40^b^	32	mixed	breast	GTV	none	mixed	N/A	43	63	83
Vassiliou et al. [16]	2007	30-40^b^	45	mixed	mixed	GTV	none	mixed	N/A	20	46	73
Vassiliou et al. [7]	2007	30-40^b^	52	mixed	mixed	GTV	by lesion type^a^	Lytic	N/A	65	134	187
								Mixed		34	66	90
								Blastic		18	32	43
Wang et al. [9]	2019	30-36/10-12	44	spine/ pelvis	breast	ROI (most representative slice)	radiated lesions	mixed	N/A	21	41.5	62
							unirradiated lesions			11	21	27
Sprave et al. [8]	2018	30/10	30 IMRT/30 3DCRT	spine	mixed	ROI	by lesion^a^ & radiation type (IMRT/3dCRT)	lytic	N/A	20/9	-1/28	N/A
								mixed		39/61	55/146	
								blastic		15/3	15/14	
Sprave et al. [20]	2018	30/10 or 24/1	27 SBRT/28 3DCRT	spine	mixed	ROI	by lesion^a^ & radiation type (SBRT/3dCRT)	all		34/33	72/41	
								lytic	N/A	54/47	86/46	N/A
Wei et al. [23]	2016	50.4/28 (abdominal RT)	42	spine (T7-L5)	Normal bone	ROI (100-120 mm^2^ placed on trabecular bone)	<5 Gy	Normal bone	N/A		-14	-19
							5-15 Gy				-28	-34
							15-25 Gy				-38	-47
							25-35 Gy				-43	-52
							>35 Gy				-40	-52
Chow et al. [14]	2004	8/1, 20/5 or 30/10	5/15/5	mixed	breast	lytic irradiated areas of most representative CT slice	8/1	lytic	N/A	28	N/A	
							20/5			41		
							30/10			45		
Eggermont et al. [6]	2016	8/1 or 20-24/5-6	42	femur	mixed	GTV+6mm	by lesion^a^ type	all	N/A	1 SF, 7 MF	N/A	
								lytic		0		
								mixed		5		
								blastic		21		
Van Wulfften Palthe et al. [10]	2018	50.4/28	21	sacrum/coccyx	chordoma	ROI (trabecular bone)	none	mixed	N/A	-37	N/A	

## Conclusions

An increase in density following radiation treatment is associated with healing. Unfortunately, from a research perspective, waiting a year is not optimal, especially when many patients with bone metastases die before then. To be feasible as an early measure, it would have to be shown that those early improvements in density translate into long-term control. There appears to be an increase in density relatively early, but studies have not been able to correlate this with later control of the disease. Our findings are limited by a lack of early imaging. Prospectively, this could be investigated in a straightforward study with regular imaging at three, six, nine, and 12 months. It would have to have a large enough enrollment to allow for the ~60% dropout rate that occurs by one year. Such a study would document that the long-term increase in density with bone cancer control can be predicted by the early changes in density.
